# Dissipation, metabolism, processing factors and risk assessment of pesticides in peaches from field cultivation to crisp processing

**DOI:** 10.1016/j.fochx.2025.102974

**Published:** 2025-08-30

**Authors:** Kai Cui, Liping Fang, Ruiyan Ding, Rui Ni, Shuai Guan, Jingyun Liang, Teng Li, Junhua Liu, Jian Wang, Zhan Dong, Xiaohu Wu, Yongquan Zheng

**Affiliations:** a Institute of Quality Standard and Testing Technology for Agro-Products, Shandong Academy of Agricultural Sciences; Shandong Provincial Key Laboratory of Test Technology on Food Quality and Safety, Jinan, Shandong 250100, People's Republic of China; b College of Food Science and Engineering, Jilin Agricultural University, Changchun, Jilin 130118, People's Republic of China; c Institute of Plant Protection, Chinese Academy of Agricultural Sciences, Beijing 100193, People's Republic of China

**Keywords:** Pesticide residue, Peach, Processing factor, Risk assessment, Peach crisps

## Abstract

Pesticide residues in peach crisps, a daily leisure snack, are insufficiently investigated. This study comprehensively explored the dissipation, metabolism, processing factors (PFs) and risk assessment of 15 commonly used pesticides in peaches from field cultivation to crisp processing. Field trial results suggest that the half-lives of the surveyed pesticides were 3.40–10.05 days in peaches. Furthermore, the metabolites clothianidin and trifloxystrobin acid were detected at concentrations of 4.28–39.79 μg/kg. All pesticide residues became concentrated in the peach crisps after freeze-drying (PF: 1.34–4.80). Contrarily, peeling and washing removed varying amounts of pesticide residues from peaches (PF: 0.076–1.24, most <1). Notably, the octanol–water partition coefficient exhibited a significant positive correlation with the crisp PFs. Dietary risk assessment results indicated that chronic and acute health risks were considered to be within acceptable limits for children and the general population. The results provide important references for promoting healthy consumption of peaches and peach crisps.

## Highlights

• The half-lives of pesticides in peaches were 3.40–10.05 days.
• The metabolites clothianidin and trifloxystrobin acid were detected.
• Pesticide residues were concentrated by 1.34–4.80 times during crisp processing.
• The physicochemical parameters of pesticides were correlated with processing factors during processing.
• Human health risks were deemed to be acceptable.

## Graphical abstract

**Figure:**
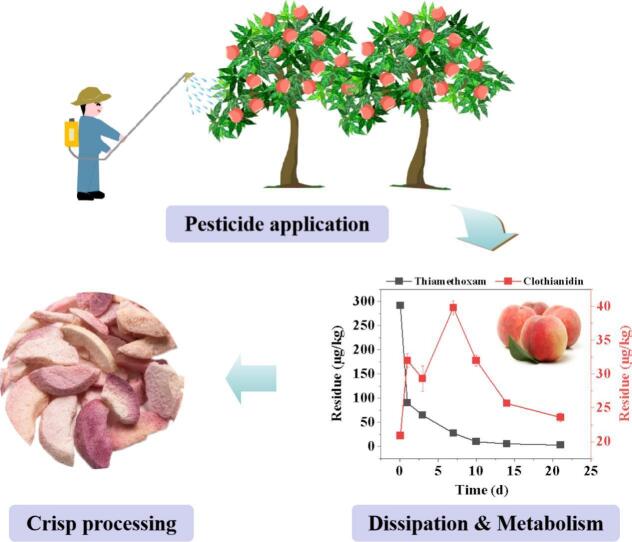

## 1 Introduction

Peaches (*Amygdalus persica* L.) are among the most popular and economic fruits worldwide owing to their excellent taste and rich nutritional content; they are rich in vitamins, sugar, fibre, minerals, organic acids and phenolic compounds (Bento et al., 2022; Kumari et al., 2023; Xie et al., 2025). In recent decades, China has steadily expanded its peach planting areas and production, ranking first in its production worldwide. In 2022, the yield reported was 1.68 × 10^7^ t (https://www.fao.org/faostat/en/#data/QCL/visualize). However, peaches ripen during hot and humid weathers, making them highly susceptible to pathogenic infections and pest infestations. Therefore, various pesticides are repeatedly sprayed on them for crop protection during peach field cultivation (Li et al., 2018; Li et al., 2023). Pesticide residues are recognized as a public health concern. Considering the high consumption of peaches, it is important to investigate the fate and dietary risks of pesticide residues to guard human health.

Studies on pesticide residues in peaches mainly focused on monitoring market samples, and they have revealed the widespread existence of pesticides in peaches, including the ones that are banned to be used on them (Choubbane et al., 2022; Jardim & Caldas, 2024; Keklik et al., 2025; Li et al., 2018). Several studies have reported the dissipation behavior and conducted risk assessment of pesticides in peaches through field experiments (Qian et al., 2023; Tian et al., 2023; Xu et al., 2021). However, these studies only covered few pesticides. Practically, many pesticides were applied on peaches to control various pests and diseases throughout their growth period. Therefore, it is important to include all the commonly used pesticides and evaluate their heath risks to humans to ensure their safe use.

In recent years, peach crisps have emerged as a new type of nutritious, healthy and leisure snack in daily life (Guo et al., 2022). Peaches are characterized by rapid ripening, softening, deterioration and decay after harvest owing to their high moisture content (Ni et al., 2023; Zhou et al., 2024). They are classified as cold-sensitive fruits and are susceptible to chilling injury and fruit flavour decline during cold storage, which influence their quality and storage life (Zhang et al., 2021). Thus, peaches are processed into crisps to facilitate their storage and maintain nutritional components. Several drying methods have been reported to produce crisp products, such as hot air drying, deep fat frying, microwave drying, explosion puffing drying and vacuum freeze-drying (VFD) (Altay et al., 2023; Wang et al., 2023). Of these, VFD is uniquely advantageous for retaining the flavour, nutrition and bioavailability of peaches to produce a high-quality snack (Zang et al., 2024). However, it is performed at a low temperature, allowing to loose water via sublimation, which may increase the pesticide residues in freeze-dried products, leading to higher dietary exposure risks (Wang et al., 2022). Existing studies mainly focused on improving the quality of peach crisps (Guo et al., 2022; Liu et al., 2021; Wang et al., 2025); however, there is little information on pesticide residue changes during peach crisp processing. Considering the potential for pesticide concentration during VFD treatment, it is imperative to comprehensively investigate the behavior of pesticide residues in peaches throughout the crisp processing.

Herein, the dissipation, metabolism, processing factors (PFs) and risks of 15 commonly used pesticides on peaches were investigated from field cultivation to crisp processing. Herein, we aimed to (1) investigate the dissipation and metabolism of pesticides in peaches under field conditions, (2) determine the residue changes of pesticides in peaches during peach crisp processing and calculate the corresponding PFs and (3) evaluate the health risks associated with peach consumption in different populations. The results of this study provide insights into the behavior of residues of commonly used pesticides during peach plantation and peach crisp processing, while supporting safe and healthy consumption of peaches and peach crisps by consumers.

## 2 Materials and methods

### 2.1 Chemicals and reagents

Individual standards of 15 pesticides (acetamiprid, afidopyropen, azoxystrobin, chlorantraniliprole, difenoconazole, flonicamid, fluopyram, imidacloprid, mefentrifluconazole, penconazole, pymetrozine, pyraclostrobin, tebuconazole, thiamethoxam and trifloxystrobin) and four metabolites (acetamiprid-*N*-desmethyl, clothianidin, fluopyram benzamide and trifloxystrobin acid), purchased from Alta Scientific Co., Ltd. (Tianjin, China), were dissolved in acetonitrile at 1000 mg/L or 100 mg/L. High-performance liquid chromatography (HPLC)-grade acetonitrile, ammonium acetate and formic acid were obtained from Fisher Scientific (Geel, Belgium), Sigma-Aldrich Chemical Co., Ltd. (Darmstadt, Germany) and Macklin Biochemical Co., Ltd. (Shanghai, China), respectively. Analytical-grade sodium chloride (NaCl), anhydrous magnesium sulfate (MgSO_4_) and ammonia solutions were acquired from Sinopharm Chemical Reagent (Shanghai, China). Nylon syringe filters (0.2 μm) and octadecylsilane (C18) were supplied by Agela Technologies (Beijing, China).

### 2.2 Field experiments and sample collection

Field experiments were conducted in the cropping season (from June to July) in 2024 in Changqing District, Jinan City, Shandong Province, China. The experimental designs consisted of two plots, one treatment plot (six trees) and another control plot (six trees). Each pesticide was applied at their highest recommended dosages and application times, and detailed information is presented in Table S1. The pesticides were uniformly mixed in water and sprayed at a 7-day interval. For the dissipation experiment, >2.0 kg of peaches were sampled at 2 h and 1, 3, 7, 10, 14 and 21 d after the final pesticide application. For the processing experiment, >20.0 kg of peaches were sampled at 1 d after the last spraying. All peach samples were immediately homogenized or processed following their transportation to the laboratory.

### 2.3 Peach processing

Peeling: Approximately 2.0 kg of peaches were peeled to obtain peel and pulp samples.

Washing: Approximately 3.0 kg of peaches were soaked in tap water and manually washed. The samples were collected at different washing time of 0.5, 1, 2 and 5 min.

Crisp processing: The peach samples were first cut into 1-cm thick slices and pre-frozed in a refrigerator at −80 °C for 12 h. Next, all frozen samples were freeze-dried using a vacuum freeze dryer (Xiaoma Mechanical Instrument Co. Ltd., Nanjing, China) at −50 °C for 60 h. Finally, the freeze-dried samples were homogenized and stored at −20 °C for further analysis.

### 2.4 Sample preparation

For pymetrozine, the homogenized samples (5 g of fresh samples or 2 g of dried samples) were placed into 50-mL polypropylene centrifuge tubes. Subsequently, 8 mL of water was added to the dried samples. Using a multi-tube vortex mixer, the mixture was extracted using 3 mL of ammonia solutions and 10 mL of acetonitrile at 2500 r/min. After shaking for 10 min, 5.0 g of NaCl was added into each tube. All tubes were shaken for 1 min and centrifuged for 5 min at 4000 r/min. Next, 5 mL of upper acetonitrile extract were purified using 150 mg of C18 and 900 mg of anhydrous MgSO_4_. The tubes were vortexed for 3 min and centrifuged for 5 min at 4000 r/min. Finally, 1 mL of upper acetonitrile extract was passed through a 0.2-μm syringe filter for further instrumental analysis.

For other analytes, 10 g of fresh samples or 2 g of dried samples were extracted using 10 mL of acetonitrile containing 1 % (*v*/v) of formic acid. The other steps were consistent with the aforementioned procedures.

### 2.5 Instrumental analysis

Nineteen analytes were qualified and quantified using an Agilent 1290 Infinity II HPLC connected to a 6460 triple quadrupole mass spectrometer (MS/MS) (Agilent, USA) with an electrospray ionization (ESI) source. A Poroshell 120 EC-C18 column (100 × 4.6 mm, i.d., 2.7 μm, Agilent) was used to separate target analytes. The column temperature maintained at 40 °C, the flow rate was set at 0.3 mL/min, and the sample was injected into 2 μL. Mobile phases consisted of acetonitrile (phase A) and water with 5-mmol/L ammonium acetate and 0.1 % formic acid (phase B). Gradient elutions were performed under the following conditions: 0 min, 10 % A; 1.5 min, 10 % A; 4 min, 80 % A; 6 min, 80 % A; 6.1 min, 10 % A and 8 min, 10 % A. Source parameters included gas temperature (250 °C), drying gas flow (14 L/min), nebulizer (35 psi), sheath gas temperature (325 °C), sheath gas flow (11 L/min), capillary voltages (4000 V for positive and 3000 V for negative) and nozzle voltages (1500 V for positive and negative). Detailed information on retention time, qualitative/quantitative ions, collision energy, fragmentor and ion polarity are listed in Table S2.

### 2.6 Health risk assessment

Herein, chronic and acute dietary risk assessments from peach consumption were conducted for children and the general population, following the methods recommended by FAO/WHO (2025). The chronic dietary risk was evaluated by calculating the estimated daily intake (EDI, μg/kg bw) and the corresponding chronic risk quotients (RQc) of each pesticide using Eqs. (1) and (2). In the acute dietary risk assessment, the estimation of short-term intake (ESTI, μg/kg bw) and acute risk quotient (RQa) were calculated using Eqs. (3) and (4).

(1) EDI=STMR×F/bw/1000

(2) RQc=EDI/ADI

(3) $$\text{ESTI}=\left[\mathrm{Ue}\times \mathrm{HR}\times v+\left(\mathrm{LP}–\mathrm{Ue}\right)\times \mathrm{HR}\right]/\mathrm{bw}/1000$$

(4) RQa=ESTI/ARfD

In the above equations, STMR and HR (μg/kg) denote the median and highest residue levels in peaches, respectively. F denotes the average peach intake (8.23 g); bw, body weight (15.5 kg for 1–6-year-olds children and 67.0 kg for >2-year-olds general population); Ue, the unit weight of peaches (255 g); LP, the large portion of peaches (306 g for children and 703.99 g for the general population) and ADI and ARfD (μg/kg bw), the acceptable daily intake and acute reference dose, respectively (Table S3). RQ > 1 indicates that human health risk is unacceptable; otherwise, the health risk is deemed to be acceptable. Notably, the metabolites, including acetamiprid-*N*-desmethyl, fluopyram benzamide and trifloxystrobin acid, were converted into parent pesticide according to the molar mass to calculate the dietary exposure risk. Furthermore, a value of half of the limit of quantification (LOQ) was assigned to the concentration that was below the LOQ while calculating.

### 2.7 Method validation

According to the European Commission SANTE/11312/2021 v2 guidelines (Pihlström et al., 2021), the proposed method was thoroughly validated for 19 analytes and key performance parameters, such as linearity, LOQ, matrix effect (ME), accuracy and precision, were assessed. Before validation, a blank sample was analyzed to confirm the absence of all target pesticides, ensuring its suitability for use in the validation process. Linearity was evaluated through solvent-based and matrix-matched calibrations across concentration ranges of 1–2000 μg/L or 5–2000 μg/L, depending on the analyte. The LOQ was recognized as the lowest validated spiked concentration in peaches that met the acceptable performance criteria. The ME, representing signal suppression or enhancement attributable to the peach matrix, was quantified using Eq. (5). To evaluate accuracy and precision, recovery studies were conducted at three concentration levels (1 or 5, 100 and 2000 μg/kg), with five replicates per level.

(5) $$\mathrm{ME}\;\left(\%\right)=\left[\text{slope}\;\left(\text{matrix}\right)/\text{slope}\;\left(\text{solvent}\right)-1\right]\times 100\%$$

In the equation, slope (matrix) and slope (solvent) are the slopes of matrix-matched and solvent standard curves, respectively.

### 2.8 Data analysis

The first-order kinetic equation was employed to assess the dissipation dynamics of pesticides in peaches using Eqs. (6) and (7):

(6) $$C_{t}=C_{0}\times e^{-\mathrm{kt}}$$

(7) $$t_{1/2}=\left(\text{ln2}\right)/k$$

In the equations, C_0_ and C_t_ (μg/kg) denote the initial residue level and residue level at time t (d), respectively; k, the dissipation rate constant; and t_1/2_ (d), the half-life.

The PF was calculated to assess the residue changes of pesticides in peaches during processing using Eq. (8):

(8) $$\mathrm{PF}=C_{\mathrm{pc}}/C_{\mathrm{rac}}$$

In the equation, C_pc_ and C_rac_ denote the residue levels in the processed and raw agricultural commodity, respectively. A PF > 1 indicates an increase in pesticide residues during processing, whereas a PF < 1 indicates a decrease in pesticide residues.

## 3 Results

### 3.1 Method validation

The data of method validation are presented in Table 1. All solvent and matrix-matched standard curves demonstrated satisfactory linearity with the correlation coefficient (R^2^) of 0.9901–1 over the concentration range of 1 or 5–2000 μg/kg. The LOQ of flonicamid was 5 μg/kg, whereas the LOQs for other analytes were 1 μg/kg. The MEs ranged from −49.01 % to 49.08 % for different analytes; therefore, matrix-matched standard curves were used to quantify all analytes to reduce the influence of ME. Accuracy and precision were evaluated using the standard recoveries and relative standard deviations (RSDs). The average recovery rates of different pesticides ranged from 76.02 % to 116.34 %, with RSDs of 0.88 %–17.83 %, thereby meeting the criteria (recovery range of 70 %–120 %, RSD < 20 %). Overall, the proposed method was suitable for the detection of the 19 analytes in peaches.

**Table 1:** Regression equations, correlation coefficient (R^2^), matrix effect (ME), average recoveries (%) and relative standard deviation (RSD, %) for different pesticides.

Pesticides	Matrix	Regression equations	R^2^	ME (%)	Average recovery, % (RSD, %)		
					1 or 5 μg/kg	100 μg/kg	2000 μg/kg
Acetamiprid	acetonitrile	y = 31,131× + 83,104	0.9986	–			
	peach	y = 27,681× + 14,357	0.9996	−11.08	100.37 (1.30)	100.18 (2.00)	100.60 (3.17)
Acetamiprid-*N*-desmethyl	acetonitrile	y = 36,294× + 98,505	0.9986	–			
	peach	y = 20,461× + 3815	1.0000	−43.62	95.49 (1.46)	95.32 (0.88)	94.39 (2.33)
Afidopyropen	acetonitrile	y = 379× + 447	0.9997	–			
	peach	y = 565× - 1217	0.9994	49.08	94.13 (10.34)	96.79 (5.00)	101.68 (17.83)
Azoxystrobin	acetonitrile	y = 81,162× + 6566	0.9995	–			
	peach	y = 87,980× + 183,760	0.9968	8.40	92.42 (2.59)	97.89 (3.72)	106.04 (5.02)
Chlorantraniliprole	acetonitrile	y = 2417× + 2209	0.9996	–			
	peach	y = 3094× + 3874	0.9960	28.01	108.64 (4.47)	103.46 (3.40)	107.60 (6.85)
Clothianidin	acetonitrile	y = 5964× + 17,361	0.9970	–			
	peach	y = 3058 + 1213	0.9997	−48.73	97.05 (1.78)	98.48 (1.49)	100.18 (2.69)
Difenoconazole	acetonitrile	y = 11,592× - 13,031	0.9998	–			
	peach	y = 16,541× - 20,167	0.9993	42.69	98.39 (3.02)	97.77 (2.01)	102.41 (2.24)
Flonicamid	acetonitrile	y = 331× + 2557	0.9901	–			
	peach	y = 256× + 6	0.9996	−22.66	97.70 (7.78)	96.80 (4.28)	96.98 (3.74)
Fluopyram	acetonitrile	y = 16,775× - 9939	0.9997	–			
	peach	y = 20,147× - 21,331	0.9990	20.10	102.34 (2.52)	101.72 (2.38)	108.95 (4.66)
Fluopyram benzamide	acetonitrile	y = 4128× + 12,205	0.9974	–			
	peach	y = 2105× + 962	0.9998	−49.01	92.88 (3.97)	95.08 (1.62)	94.26 (2.59)
Imidacloprid	acetonitrile	y = 5509× + 40,684	0.9935	–			
	peach	y = 4854× + 22,330	0.9998	−11.89	97.58 (3.17)	102.59 (2.42)	104.34 (1.63)
Mefentrifluconazole	acetonitrile	y = 8136× - 9399	0.9996	–			
	peach	y = 10,771× - 502	0.9997	32.39	103.67 (1.35)	101.60 (3.11)	108.60 (3.99)
Penconazole	acetonitrile	y = 13,837× - 5182	0.9997	–			
	peach	y = 16,789× + 9197	0.9999	21.33	108.49 (1.20)	109.13 (2.05)	116.34 (2.79)
Pymetrozine	acetonitrile	y = 20,910× + 75,817	0.9976	–			
	peach	y = 15,973× - 110	1.0000	−23.61	78.73 (4.47)	76.32 (3.45)	76.02 (4.01)
Pyraclostrobin	acetonitrile	y = 12,816× + 12,850	0.9997	–			
	peach	y = 15,495× + 7035	0.9999	20.90	99.26 (4.87)	99.30 (4.34)	103.68 (3.41)
Tebuconazole	acetonitrile	y = 18,011× - 9334	0.9998	–			
	peach	y = 22,219× - 1390	0.9997	23.36	106.05 (3.34)	102.51 (2.33)	103.62 (4.37)
Thiamethoxam	acetonitrile	y = 14,302× + 78,029	0.9938	–			
	peach	y = 12,516× + 12,139	0.9998	−12.49	97.04 (3.50)	100.35 (1.71)	102.27 (1.01)
Trifloxystrobin	acetonitrile	y = 30,308× - 47,941	0.9992	–			
	peach	y = 36,128× - 38,334	0.9994	19.20	104.27 (2.57)	101.18 (1.86)	105.60 (3.09)
Trifloxystrobin acid	acetonitrile	y = 5640× - 7896	0.9998	–			
	peach	y = 7484× + 10,486	0.9994	32.70	92.76 (2.41)	94.19 (3.26)	104.66 (8.46)

### 3.2 Dissipation and metabolism of pesticides in peaches in the field

The dissipation of pesticide residues under peach field cultivation is presented in Tables 2 and S4. For the 15 parent pesticides, the initial concentrations (2 h after spraying) were in the range of 27.00–1239.67 μg/kg, which gradually decreased with extending sampling points. At the end of the experiment (21 d after spraying), the degradation rates of the 15 pesticides reached 78.83 %–98.88 %. The decrease in pesticide concentration followed the first-order kinetic equation, with an R^2^ of 0.7548–0.9935, and the pesticides' half-lives ranged from 3.40 to 10.05 d in peaches. The metabolites acetamiprid-*N*-desmethyl and fluopyram benzamide (acetamiprid and fluopyram metabolites, respectively) were not detected in peaches under field conditions; however, clothianidin and trifloxystrobin acid (thiamethoxam and trifloxystrobin metabolites, respectively) were detected in all peach samples at concentrations of 20.89–39.79 μg/kg and 4.28–19.44 μg/kg, respectively. Furthermore, the residual amounts of these two detected metabolites first increased to a peak and then gradually kept deceasing throughout the sampling period.

**Table 2:** Dissipation dynamic, correlation coefficient (R2) and half-lives of different pesticides in peaches under field conditions.

Pesticides	Initial deposition (μg/kg)	Dynamic equations	R^2^	Half lives (d)
Acetamiprid	138.43	y = 80.76e^-0.085x^	0.8356	8.15
Afidopyropen	27.00	y = 14.80e^-0.118x^	0.8363	5.87
Azoxystrobin	576.21	y = 530.36e^-0.142x^	0.9523	4.88
Chlorantraniliprole	477.26	y = 476.03e^-0.069x^	0.9785	10.05
Difenoconazole	718.81	y = 637.09e^-0.099x^	0.9787	7.00
Flonicamid	367.71	y = 238.78e^-0.088x^	0.8982	7.88
Fluopyram	1239.67	y = 789.42e^-0.095x^	0.8747	7.30
Imidacloprid	1178.45	y = 634.62e^-0.116x^	0.8776	5.98
Mefentrifluconazole	1019.85	y = 930.94e^-0.094x^	0.9673	7.37
Penconazole	480.64	y = 400.93e^-0.175x^	0.9871	3.96
Pymetrozine	77.42	y = 23.72e^-0.158x^	0.7548	4.39
Pyraclostrobin	917.92	y = 843.72e^-0.088x^	0.9841	7.88
Tebuconazole	808.38	y = 537.73e^-0.131x^	0.9773	5.29
Thiamethoxam	291.38	y = 134.55e^-0.204x^	0.9103	3.40
Trifloxystrobin	1136.72	y = 1071.76^e-0.126x^	0.9935	5.50

### 3.3 Residue changes of pesticides during peach processing

**Fig. 1:**
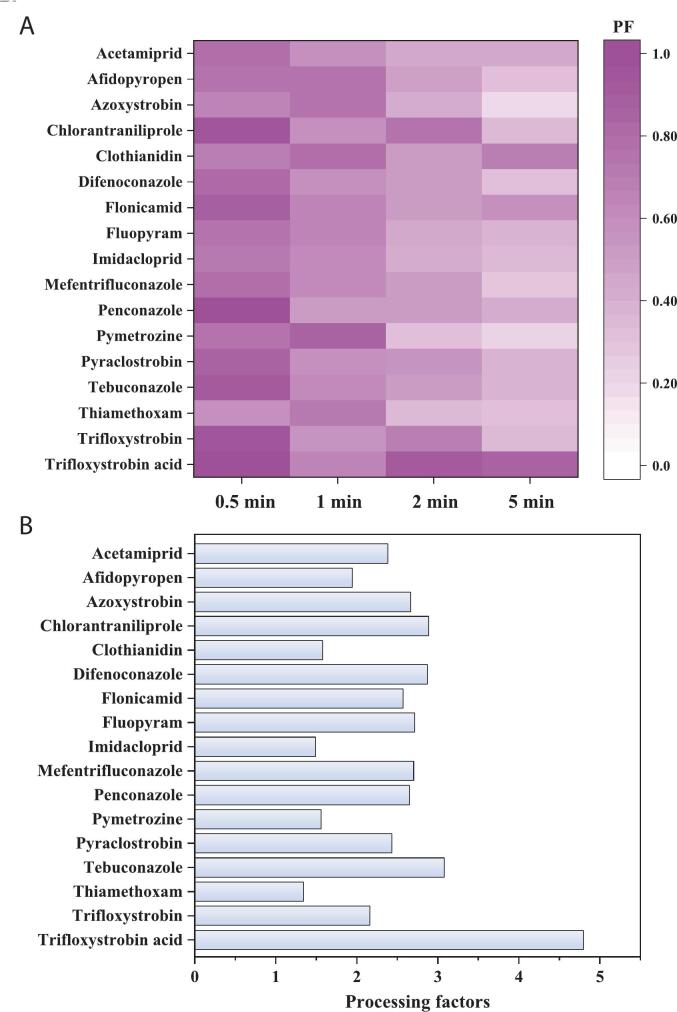
The processing factors (PFs) of pesticides in peaches during washing (A) and crisp processing (B).

Herein, the residue changes of 15 parent pesticides and two metabolites (clothianidin and trifloxystrobin acid) were investigated during peach processing, including peeling, washing and crisp processing. The residues of the surveyed pesticides in peach peels were 1.04–2.67 times and 1.35–32.66 times higher than those in whole peaches and peach pulps, respectively. Peeling treatment removed 3.08 %–92.43 % of pesticide residues from peaches with PFs ranging from 0.076 to 0.97 (Table S5). For washing treatment, different washing times were applied to remove varying amounts of pesticide residues from peaches with PFs of <1 (0.17–0.97), except for trifloxystrobin acid (PF = 1.24) with 0.5 min washing time (Fig. 1A). Generally, better removal efficiency of pesticide residues from peaches can be achieved with prolonged washing time. The PFs of the peach crisps ranged from 1.34 to 4.80 (Fig. 1B), indicating that the freeze-drying process markedly increased pesticide residues in peaches during crisp processing. Notably, fluopyram benzamide was not detected in all field samples but was detected in peach peels and crisps at concentrations of 3.50 and 10.79 μg/kg, respectively.

**Fig. 2:**
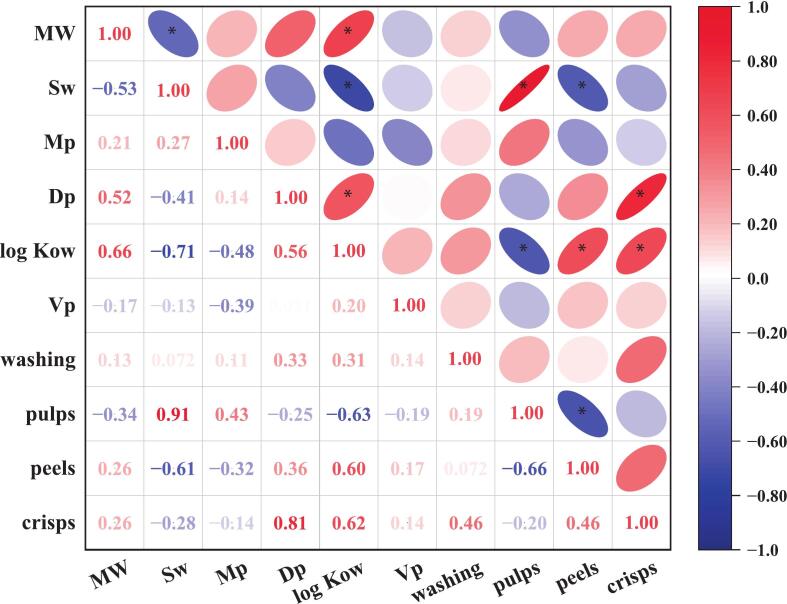
Heatmap based on Pearson's correlation coefficients showing the associations between the PFs and physicochemical properties of different pesticides (molecular weight, MW; solubility in water, Sw; melting point, Mp; degradation point, Dp; octanol–water partition coefficient, log Kow; vapour pressure, Vp).

The relationships between pesticide physicochemical properties (molecular weight, MW; solubility in water, Sw; melting point, Mp; degradation point, Dp; octanol–water partition coefficient, log Kow and vapour pressure, Vp) (Table S6) and average PFs of different processing treatments (peeling, washing and crisp processing) were evaluated using Pearson's correlation coefficients, as presented in Fig. 2. The PFs of pulps showed a significantly positive correlation with Sw and a negative correlation with log Kow. Contrarily, the PFs of peels exhibited a strong negative correlation with Sw and a positive correlation with log Kow. This suggests that pesticides with higher Sw and lower log Kow were more likely to penetrate peach pulps through their peels. A significantly positive correlation was observed between crisp PFs as well as Dp and log Kow, implying that pesticides with higher Dp and log Kow were more likely to remain in peach crisps.

### 3.4 Dietary risk assessment of pesticides in peaches

Maximum residue limits (MRLs) are the standard for evaluating pesticide residue safety in food. Table S4 presents the MRLs of different pesticides in peaches from various countries, including China and the USA, along with organizations such as Codex Alimentarius Commission (CAC) and European Union (EU). Herein, we measured pesticide concentrations in peaches at different growth stages (2 h and 1, 3, 7, 10, 14 and 21 d after application). For acetamiprid, azoxystrobin, chlorantraniliprole, clothianidin, flonicamid, mefentrifluconazole and trifloxystrobin, all detected residues were in lower concentrations than the MRLs established by China, the CAC, the USA and EU. Although the residue levels of some pesticides exceeded the MRLs established by certain countries during their initial application period, the residues at the recommended pre-harvest intervals were below their corresponding MRLs, with the exception of imidacloprid residues (73.5 μg/kg), which exceeded the MRL established by the EU (10 μg/kg).

**Fig. 3:**
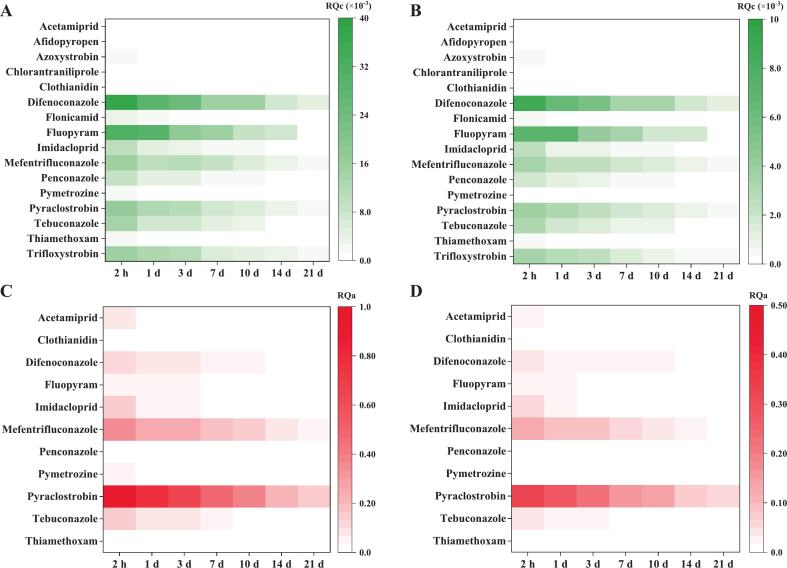
Heatmaps of the chronic and acute risk quotient (RQc and RQa, respectively) values of different pesticides for children (A, C) and the general population (B, D) from peach consumption.

Chronic and acute dietary risks were evaluated based on the pesticide residues in peaches at different sampling intervals and peach consumption data in different populations. The results of dietary risk assessment for different pesticides are summarized in Fig. 3 and Table S7. To evaluate chronic dietary risks, the calculated RQc for children and the general population were 1.33 × 10^−5^ − 0.0044 and 3.08 × 10^−6^ − 0.0010 for intervals of 21 d, 1.39 × 10^−5^ − 0.0076 and 3.22 × 10^−6^ − 0.0018 for 14 d, 2.06 × 10^−5^ − 0.015 and 4.78 × 10^−6^ − 0.0034 for 10 d, 3.26 × 10^−5^ − 0.016 and 7.54 × 10^−6^ − 0.0037 for 7 d, 7.14 × 10^−5^ − 0.024 and 1.65 × 10^−5^ − 0.0056 for 3 d, 7.45 × 10^−5^ − 0.031 and 1.72 × 10^−5^ − 0.0071 for 1 d and 1.11 × 10^−4^ − 0.038 and 2.57 × 10^−5^ − 0.0088 for 2 h, respectively, which were much less than 1 (Fig. 3A, B). In the acute dietary risk assessment, afidopyropen, azoxystrobin, chlorantraniliprole, flonicamid and trifloxystrobin were not considered owing to their unnecessary or unavailable ARfD values. For the other pesticides, the RQa values for children and the general population were 1.11 × 10^−4^ − 0.15 and 3.81 × 10^−5^ − 0.052 for 21 d, 2.92 × 10^−4^ − 0.22 and 1.01 × 10^−4^ − 0.077 for 14 d, 5.35 × 10^−4^ − 0.39 and 1.84 × 10^−4^ − 0.13 for 10 d, 0.0015–0.47 and 5.03 × 10^−4^ − 0.16 for 7 d, 0.0026–0.66 and 8.85 × 10^−4^ − 0.23 for 3 d, 0.0028–0.78 and 9.66 × 10^−4^ − 0.27 for 1 d and 0.0018–0.97 and 6.31 × 10–04 − 0.33 for 2 h, respectively, which were smaller than 1 (Fig. 3C, D). Notably, pyraclostrobin demonstrated the highest RQa value for children and the general population at each sampling interval (0.15–0.97 and 0.052–0.33, respectively). Overall, the chronic and acute dietary risks of exposure to the surveyed pesticides through peach consumption were within acceptable limits.

## 4 Discussion

Understanding the dissipation behavior and half-lives of pesticides is essential for evaluating their persistence and predicting their residue changes in crops, which guides the rational use of pesticides and ensures food safety (Fantke et al., 2014; Pan et al., 2024). The results of the present study suggest that the half-lives of the tested pesticides were within 3.40–10.05 d, which showed low persistence and fast dissipation (<16-d half-life) (Li et al., 2021). Zhang et al. (2022) reported that the half-life range of flonicamid in peaches was 3.44–7.63 d, which was shorter than the one obtained from our experimental results (7.88 d). Contrarily, the half-lives of thiamethoxam in peaches (4.9–5.5 d) reported by Tian et al. (2022) were longer than those observed in our study (3.40 d). Similarly, Dong et al. (2019) found that the dissipation half-life of difenoconazole in peaches was 4.4–13 d, which is consistent with our results (7.00 d). Various factors influence pesticide dissipation in peaches, such as pesticide properties, pesticide application dosages and time, environmental factors (temperature, moisture and solar radiation) and crop varieties (Farha et al., 2016; Li et al., 2024). Furthermore, as peach fruits gradually increase in weight and surface area during growth, the dilution effect may play an important role in reducing pesticide residues (Li, 2023). Some metabolites, such as clothianidin and trifloxystrobin acid, were detected in peaches at concentrations of 4.28–39.79 μg/kg. Similar to our results, previous studies confirmed their presence in peaches and other crops following parent pesticide application (Cui et al., 2023; Mandal et al., 2023; Tian et al., 2022). Given their inherent toxicity and potential effects when combined with parent pesticides, these metabolites need continuous monitoring and evaluation.

Peach crisps have emerged as a popular leisure snack in daily life. Many studies have investigated pesticide residues in peaches (Choubbane et al., 2022; Jardim & Caldas, 2024; Keklik et al., 2025) but have disregarded the pesticide residues in peach crisps. The results of our study indicated that the residues of pesticides, including the detected metabolites, were concentrated by 1.34–4.80 times during crisp processing using the VFD method. Many previous studies have confirmed that the pesticide residues increased in the processed commodities following the VFD treatment (Rutkowska et al., 2023; Wang et al., 2022). Water sublimation may explain the increase in pesticide residues, whereas pesticide degradation and evaporation may be attributed to their decrease (Cui, Wang, Guan, et al., 2024), and both of them jointly decide the concentration or dilution effect of pesticide residues in processed commodities. Moreover, our results suggest that pesticides with higher log Kow value are more likely to remain in peach crisps following processing. Log Kow value determines the polarity of the compounds, which is closely associated with the transfer capability of pesticides (López-Blanco et al., 2018). Pesticides with higher log Kow are typically characterized by greater lipophilicity and hydrophobicity (Cui, Wang, Ma, et al., 2024). Therefore, they are less soluble in water for sublimation, resulting at higher residue levels in peach crisps. Our results suggest that peeling and washing led to the removal of varying amounts of pesticide residues from peaches. In addition, the pesticides with higher Sw and lower log Kow are more likely to penetrate peach pulps through the peels, making their removal difficult through peeling and washing. Similarly, previous studies have concluded that pesticides with low lipophilicity (log Kow < 2) tend to more readily migrate into the pulp and residues in this fraction are not easily removed through peeling (Liu et al., 2023). Various studies have confirmed that these two processing treatments are technically simple, quick and cost-effective for the removal of pesticide residues from different food types (Cámara et al., 2020; Gao et al., 2024; Pan et al., 2024; Tian et al., 2023).

The results of chronic and acute dietary risk assessment indicated that the assessed peach pesticides were within acceptable limits for children and the general population as the RQc (3.08 × 10^−6^ − 0.0088) and RQa (3.81 × 10^−5^ − 0.97) values were < 1. Similarly, several studies reported that dietary exposure to pesticides from peach consumption posed no health risks based on the field data (Qian et al., 2023; Xu et al., 2021). Herein, pyraclostrobin posed a higher acute dietary risk than other pesticides, owing to its higher concentration in peaches and lower ARfD value (50 μg/kg bw). Despite the acceptable RQ values, the health risks due to peach consumption should not be ignored. First, many pesticides are often simultaneously or successively used to control multiple peach diseases and insect pests during the growing season and co-existing pesticide residues in peaches may pose cumulative toxicity to humans (Yang et al., 2024). Moreover, under biological and abiotic factors, certain pesticides in peaches can induce the production of toxicological metabolites in humans that may synergistically act with the parent compounds (Treviño et al., 2023). Second, pesticide residue levels may increase during post-processing treatments, such as crisp processing, increasing the dietary exposure risks. Therefore, pesticide residues in peaches and their processed products should be carefully monitored to guard human health and ensure food safety.

## 5 Conclusions

To our knowledge, this study is the first to comprehensively evaluate the dissipation, metabolism, PFs and risk assessment of 15 commonly used pesticides in peaches from field cultivation to crisp processing. Field experiments suggest that the dissipation of all pesticides followed the first-order kinetic equation, with half-lives of 3.40–10.05 d. Furthermore, the metabolites clothianidin and trifloxystrobin acid were detected in peaches under field conditions at concentrations of 4.28–39.79 μg/kg. Notably, the pesticide residue levels were found 1.34–4.80 times higher in peach crisps than those in raw peaches, indicating high dietary risks for humans. Contrarily, different amounts of pesticide residues were removed from peaches through washing or peeling. The pesticide residues in peaches during processing were significantly related to some pesticide physicochemical properties, of which log Kow demonstrated a significantly positive correlation with the crisp PFs. The results of chronic and acute risk assessment indicated that the health risks of dietary exposure to the surveyed pesticides through peach consumption were within acceptable limits. Notably, more attention should be paid to pyraclostrobin owing to its higher acute risk values. Our study demonstrated the dissipation behavior of multiple pesticides in peaches under field conditions, and the pesticide residue changes during crisp processing, offering reliable baseline data for their rational use and for promoting healthy consumption.

## CRediT authorship contribution statement

**Kai Cui:** Writing – review & editing, Writing – original draft, Software, Resources, Methodology, Funding acquisition, Formal analysis, Data curation. **Liping Fang:** Methodology, Investigation. **Ruiyan Ding:** Formal analysis, Data curation. **Rui Ni:** Resources. **Shuai Guan:** Software. **Jingyun Liang:** Resources. **Teng Li:** Investigation. **Junhua Liu:** Resources. **Jian Wang:** Writing – review & editing, Conceptualization. **Zhan Dong:** Writing – review & editing, Funding acquisition, Conceptualization. **Xiaohu Wu:** Validation, Supervision. **Yongquan Zheng:** Validation, Supervision.

## Declaration of competing interest

The authors declare that they have no known competing financial interests or personal relationships that could have appeared to influence the work reported in this paper.

## Data Availability

No data was used for the research described in the article.

## Appendix A Supplementary data

Supplementary material Image 1
