# Supplementary material for: Dissipation, metabolism, processing factors and risk assessment of pesticides in peaches from field cultivation to crisp processing
Source: Food Chem X. 2025 Aug 30;30:102974. doi: 10.1016/j.fochx.2025.102974 (PMC12445690; doi:10.1016/j.fochx.2025.102974)
Supplement: Supplementary file 1 — Supplementary material [file mmc1.docx]

**Dissipation, metabolism, processing factors and risk assessment of pesticides in peaches from field cultivation to crisp processing**

Kai Cui^a^, Liping Fang^a^, Ruiyan Ding^a^, Rui Ni^a^**^,^** ^b^, Shuai Guan^a^, Jingyun Liang^a^, Teng Li^a^, Junhua Liu^a^, Jian Wang^a^**^,^ ***, Zhan Dong^a^**^,^ ***, Xiaohu Wu^c^, Yongquan Zheng^c^

^a^ Institute of Quality Standard and Testing Technology for Agro-Products, Shandong Academy of Agricultural Sciences; Shandong Provincial Key Laboratory of Test Technology on Food Quality and Safety, Jinan, Shandong, 250100, People’s Republic of China

^b^ College of Food Science and Engineering, Jilin Agricultural University, Changchun, Jilin, 130118, People’s Republic of China

^c^ Institute of Plant Protection, Chinese Academy of Agricultural Sciences, Beijing, 100193, People’s Republic of China

*** Corresponding authors:**

E-mail: [zhandongsaas@163.com](mailto:zhandongsaas@163.com) (Zhan Dong); [Jianwangsaas@163.com](mailto:Jianwangsaas@163.com) (Jian Wang)

**Table captions**

**Table S1.** Spray application parameters of 11 commercial pesticide products on peaches.

**Table S2.** Optimized HPLC–MS/MS parameters and retention time of different pesticides.

**Table S3.** Acceptable daily intake (ADI) and acute reference dose (ARfD) of different pesticides.

**Table S4.** Residue levels, degradation rates and the maximum residue limits (MRLs) of different pesticides in peaches.

**Table S5.** Residue levels and processing factors (PFs) of different pesticides during peach processing.

**Table S6.** Physicochemical parameters of different pesticides used in the study (molecular weight, MW; solubility in water, Sw, 20°C, mg/ L; melting point, Mp, °C; degradation point, Dp, °C; octanol–water partition coefficient, logKow, pH = 7, 20°C; vapour pressure, 20°C, mPa, respectively).

**Table S7.** The estimated daily intake (EDI), estimation of short-term intake (ESTI), chronic risk quotient (RQc) and acute risk quotient (RQa) values of different pesticides for children and the general populations from peach consumption.

**Table S1.** Spray application parameters of 11 commercial pesticide products on peaches.

| Pesticides | Formulation | Contents | Application dosages/ dilution multiple | Application volume | Application times | Application interval (d) | Pre-harvest interval (d) |
| --- | --- | --- | --- | --- | --- | --- | --- |
| Acetamiprid and flonicamid | water dispersible granule | 46%  (acetamiprid, 12%; flonicamid, 34%) | 8000 | 2 L/tree | 2 | 7 | 14 |
| Afidopyropen | dispersible concentrate | 50 g/L | 8000 | 2 L/tree | 2 | 7 | 14 |
| Azoxystrobin and difenoconazole | suspension concentrate | 325 g/L  (azoxystrobin, 200 g/L; difenoconazole 125 g/L) | 1500 | 2 L/tree | 3 | 7 | 14 |
| Chlorantraniliprole | suspension concentrate | 200 g/L | 4000 | 2 L/tree | 2 | 7 | 7 |
| Fluopyram and trifloxystrobin | suspension concentrate | 43%  (fluopyram, 21.5%; trifloxystrobin, 21.5%) | 1500 | 2 L/tree | 2 | 7 | 14 |
| Imidacloprid | water dispersible granule | 70% | 5000 | 2 L/tree | 1 | - | 21 |
| Mefentrifluconazole and pyraclostrobin | suspension concentrate | 400 g/L  (pyraclostrobin, 200 g/L; mefentrifluconazole, 200 g/L) | 1500 | 2 L/tree | 3 | 7 | 14 |
| Penconazole | emulsion in water | 10% | 1500 | 2 L/tree | 2 | 7 | 14 |
| Pymetrozine | wettable powder | 25% | 1250 | 2 L/tree | 1 | - | 14 |
| Tebuconazole | suspension concentrate | 430 g/L | 3000 | 2 L/tree | 2 | 7 | 21 |
| Thiamethoxam | water dispersible granule | 25% | 6000 | 2 L/tree | 3 | 7 | 10 |

**Table S2.** Optimized HPLC–MS/MS parameters and retention time of different pesticides.

| Pesticides | Retention time  (min) | Quantitative ion pair  (m/z) | Qualitative ion pair  (m/z) | Fragmentor  (V) | Collision Energy  (V) | Ion polarity |
| --- | --- | --- | --- | --- | --- | --- |
| Acetamiprid | 3.829 | 223.08/126.1 | 223.08/73 | 166 | 20/72 | Positive |
| Afidopyropen | 4.605 | 594.1/148 | 594.1/202 |  | 60/40 | Positive |
| Azoxystrobin | 4.908 | 404.13/372 | 404.13/344.1 |  | 12/24 | Positive |
| Chlorantraniliprole | 4.701 | 483.9/452.8 | 483.9/285.9 |  | 20/20 | Positive |
| Difenoconazole | 5.318 | 406.07/251 | 406.07/337 |  | 28/16 | Positive |
| Flonicamid | 3.403 | 230/203.1 | 230/174.1 |  | 13/17 | Positive |
| Fluopyram | 4.989 | 397/173 | 397/207.9 |  | 40/20 | Positive |
| Imidacloprid | 3.717 | 256.06/175.1 | 256.06/209 |  | 20/12 | Positive |
| Mefentrifluconazole | 5.051 | 398.1/70 | 398.1/182.1 |  | 50/35 | Positive |
| Penconazole | 5.135 | 284.1/70.3 | 284.1/159.2 |  | 18/30 | Positive |
| Pymetrozine | 0.912 | 218.1/105 | 218.1/78 |  | 15/50 | Positive |
| Pyraclostrobin | 5.385 | 388/163 | 388/194 |  | 20/20 | Positive |
| Tebuconazole | 5.023 | 308.1/70 | 308.1/150.9 |  | 20/30 | Positive |
| Thiamethoxam | 3.928 | 292.03/211 | 292.03/181 |  | 8/24 | Positive |
| Trifloxystrobin | 5.553 | 409.14/186 | 409.14/145 |  | 20/60 | Positive |
| Metabolites |  |  |  |  |  |  |
| Acetamiprid-N-desmethyl | 3.657 | 209.06/126 | 209.06/90 |  | 16/36 | Positive |
| Clothianidin | 3.623 | 250.02/169.1 | 250.02/132.1 |  | 12/16 | Positive |
| Fluopyram benzamide | 3.564 | 190/130 | 190/102 |  | 20/35 | Positive |
| Trifloxystrobin acid | 4.974 | 395/186 | 395/148 |  | 19/11 | Positive |

**Table S3.** Acceptable daily intake (ADI) and acute reference dose (ARfD) of different pesticides.

| Pesticides | ADI  (μg/kg bw) | ARfD  (μg/kg bw) |
| --- | --- | --- |
| Acetamiprid | 70 | 100 |
| Afidopyropen | 80 | not found |
| Azoxystrobin | 200 | unnecessary |
| Chlorantraniliprole | 2000 | unnecessary |
| Clothianidin | 100 | 600 |
| Difenoconazole | 10 | 300 |
| Flonicamid | 70 | unnecessary |
| Fluopyram | 10 | 500 |
| Imidacloprid | 60 | 400 |
| Mefentrifluconazole | 35 | 150 |
| Penconazole | 30 | 800 |
| Pymetrozine | 30 | 100 |
| Pyraclostrobin | 30 | 50 |
| Tebuconazole | 30 | 300 |
| Thiamethoxam | 80 | 1000 |
| Trifloxystrobin | 40 | unnecessary |

**Table S4.** Residue levels, degradation rates and the maximum residue limits (MRLs) of different pesticides in peaches.

| Pesticides | Time | Residues (μg/kg) | Degradation rates (%) | MRL (μg/kg) | | | |
| --- | --- | --- | --- | --- | --- | --- | --- |
|  |  |  |  | China | CAC | USA | EU |
| Acetamiprid | 2 h | 138.43 ± 2.47 | - | 2000 | 700 | 1500 | 200 |
|  | 1 d | 61.48 ± 1.4 | 55.59 |  |  |  |  |
|  | 3 d | 54.32 ± 1.55 | 60.76 |  |  |  |  |
|  | 7 d | 35.65 ± 0.79 | 74.25 |  |  |  |  |
|  | 10 d | 31.78 ± 0.51 | 77.04 |  |  |  |  |
|  | **14 d** | **21.28 ± 0.09** | 84.63 |  |  |  |  |
|  | 21 d | 17.31 ± 0.42 | 87.5 |  |  |  |  |
| Afidopyropen | 2 h | 27 ± 1.99 | - | - | 10 | 30 | - |
|  | 1 d | 11.23 ± 0.27 | 58.41 |  |  |  |  |
|  | 3 d | 10.75 ± 0.51 | 60.18 |  |  |  |  |
|  | 7 d | 4.91 ± 0.4 | 81.82 |  |  |  |  |
|  | 10 d | 3.11 ± 0.14 | 88.48 |  |  |  |  |
|  | **14 d** | **2.1 ± 0.17** | 92.23 |  |  |  |  |
|  | 21 d | 2.01 ± 0.07 | 92.56 |  |  |  |  |
| Azoxystrobin | 2 h | 576.21 ± 2.48 | - | 2000 | 2000 | 1500 | 2000 |
|  | 1 d | 398.57 ± 10.06 | 30.83 |  |  |  |  |
|  | 3 d | 367.65 ± 12.93 | 36.2 |  |  |  |  |
|  | 7 d | 247.92 ± 17.91 | 56.97 |  |  |  |  |
|  | 10 d | 124.86 ± 1.35 | 78.33 |  |  |  |  |
|  | **14 d** | **46.04 ± 2.59** | 92.01 |  |  |  |  |
|  | 21 d | 33.95 ± 1.71 | 94.11 |  |  |  |  |
| Chlorantraniliprole | 2 h | 477.26 ± 47.62 | - | 2000 | 1000 | 4000 | 1000 |
|  | 1 d | 416.88 ± 51.56 | 12.65 |  |  |  |  |
|  | 3 d | 372.56 ± 39.38 | 21.94 |  |  |  |  |
|  | **7 d** | **291.41 ± 25.7** | 38.94 |  |  |  |  |
|  | 10 d | 269.1 ± 32 | 43.62 |  |  |  |  |
|  | 14 d | 195.99 ± 19.28 | 58.93 |  |  |  |  |
|  | 21 d | 101.02 ± 17.11 | 78.83 |  |  |  |  |
| Difenoconazole | 2 h | 718.81 ± 7.92 | - | 500 | 1500 | 2500 | 500 |
|  | 1 d | 527.73 ± 20.39 | 26.58 |  |  |  |  |
|  | 3 d | 453.1 ± 46.25 | 36.96 |  |  |  |  |
|  | 7 d | 298.44 ± 32.66 | 58.48 |  |  |  |  |
|  | 10 d | 276.74 ± 4.3 | 61.5 |  |  |  |  |
|  | **14 d** | **140.13 ± 10.48** | 80.51 |  |  |  |  |
|  | 21 d | 83.39 ± 2.07 | 88.4 |  |  |  |  |
| Flonicamid | 2 h | 367.71 ± 13.72 | - | 700 | 700 | 1500 | 400 |
|  | 1 d | 187.14 ± 3.08 | 49.11 |  |  |  |  |
|  | 3 d | 164.9 ± 2.83 | 55.16 |  |  |  |  |
|  | 7 d | 105.76 ± 1.66 | 71.24 |  |  |  |  |
|  | 10 d | 89.56 ± 3.11 | 75.64 |  |  |  |  |
|  | **14 d** | **65.2 ± 0.73** | 82.27 |  |  |  |  |
|  | 21 d | 44.16 ± 1.68 | 87.99 |  |  |  |  |
| Fluopyram | 2 h | 1239.67 ± 24.62 | - | 1000 | 1000 | 1000 | 1500 |
|  | 1 d | 582.71 ± 7.4 | 52.99 |  |  |  |  |
|  | 3 d | 575.24 ± 27.62 | 53.6 |  |  |  |  |
|  | 7 d | 343.74 ± 18.5 | 72.27 |  |  |  |  |
|  | 10 d | 291.69 ± 4.76 | 76.47 |  |  |  |  |
|  | **14 d** | **156.85 ± 5.13** | 87.35 |  |  |  |  |
|  | 21 d | 142.6 ± 4.67 | 88.5 |  |  |  |  |
| Imidacloprid | 2 h | 1178.45 ± 17.42 | - | 500 | 1500 | 3000 | 10 |
|  | 1 d | 473.1 ± 3.71 | 59.85 |  |  |  |  |
|  | 3 d | 365.58 ± 16.02 | 68.98 |  |  |  |  |
|  | 7 d | 219.76 ± 10.85 | 81.35 |  |  |  |  |
|  | 10 d | 175.92 ± 3.9 | 85.07 |  |  |  |  |
|  | 14 d | 104.65 ± 4.11 | 91.12 |  |  |  |  |
|  | **21 d** | **73.5 ± 1.97** | 93.76 |  |  |  |  |
| Mefentrifluconazole | 2 h | 1019.85 ± 36.49 | - | - | 2000 | 1500 | 2000 |
|  | 1 d | 701.67 ± 32.11 | 31.2 |  |  |  |  |
|  | 3 d | 707.41 ± 71.67 | 30.64 |  |  |  |  |
|  | 7 d | 541.76 ± 54.11 | 46.88 |  |  |  |  |
|  | 10 d | 403.9 ± 3.64 | 60.4 |  |  |  |  |
|  | **14 d** | **208.39 ± 10.7** | 79.57 |  |  |  |  |
|  | 21 d | 133.83 ± 3.96 | 86.88 |  |  |  |  |
| Penconazole | 2 h | 480.64 ± 5.81 | - | 100 | 80 | - | 150 |
|  | 1 d | 270.32 ± 10.92 | 43.76 |  |  |  |  |
|  | 3 d | 228.42 ± 23.33 | 52.48 |  |  |  |  |
|  | 7 d | 118.98 ± 6.81 | 75.25 |  |  |  |  |
|  | 10 d | 81.91 ± 3.62 | 82.96 |  |  |  |  |
|  | **14 d** | **29.51 ± 2.94** | 93.86 |  |  |  |  |
|  | 21 d | 10.42 ± 0.22 | 97.83 |  |  |  |  |
| Pymetrozine | 2 h | 77.42 ± 0.46 | - | 500 | - | - | 20 |
|  | 1 d | 17.81 ± 4 | 77 |  |  |  |  |
|  | 3 d | 8.66 ± 0.38 | 88.81 |  |  |  |  |
|  | 7 d | 6.13 ± 1.2 | 92.09 |  |  |  |  |
|  | 10 d | 2.67 ± 0.27 | 96.55 |  |  |  |  |
|  | **14 d** | **1.74 ± 0.3** | 97.76 |  |  |  |  |
|  | 21 d | 1.74 ± 0.17 | 97.75 |  |  |  |  |
| Pyraclostrobin | 2 h | 917.92 ± 32.74 | - | 1000 | 300 | 2500 | 300 |
|  | 1 d | 745.01 ± 15.38 | 18.84 |  |  |  |  |
|  | 3 d | 629.88 ± 59.6 | 31.38 |  |  |  |  |
|  | 7 d | 444.86 ± 57.59 | 51.54 |  |  |  |  |
|  | 10 d | 371.89 ± 11.42 | 59.49 |  |  |  |  |
|  | **14 d** | **212.02 ± 15.03** | 76.9 |  |  |  |  |
|  | 21 d | 144.08 ± 3 | 84.3 |  |  |  |  |
| Tebuconazole | 2 h | 808.38 ± 18.48 | - | 2000 | 2000 | 2000 | 600 |
|  | 1 d | 410.85 ± 4.32 | 49.18 |  |  |  |  |
|  | 3 d | 388.7 ± 17.29 | 51.92 |  |  |  |  |
|  | 7 d | 226.44 ± 12.95 | 71.99 |  |  |  |  |
|  | 10 d | 176.09 ± 4.77 | 78.22 |  |  |  |  |
|  | 14 d | 69.36 ± 2.98 | 91.42 |  |  |  |  |
|  | **21 d** | **34.79 ± 0.44** | 95.7 |  |  |  |  |
| Thiamethoxam | 2 h | 291.38 ± 5.01 | - | 1000 | 1000 | 500 | 70 |
|  | 1 d | 90.36 ± 0.39 | 68.99 |  |  |  |  |
|  | 3 d | 64.77 ± 4.23 | 77.77 |  |  |  |  |
|  | 7 d | 27.77 ± 1.51 | 90.47 |  |  |  |  |
|  | **10 d** | **10.16 ± 0.17** | 96.51 |  |  |  |  |
|  | 14 d | 5.55 ± 0.24 | 98.09 |  |  |  |  |
|  | 21 d | 3.25 ± 0.1 | 98.88 |  |  |  |  |
| Trifloxystrobin | 2 h | 1136.72 ± 28.85 | - | 3000 | 3000 | - | 3000 |
|  | 1 d | 885.72 ± 29.78 | 22.08 |  |  |  |  |
|  | 3 d | 797.49 ± 60.19 | 29.84 |  |  |  |  |
|  | 7 d | 433.75 ± 30.27 | 61.84 |  |  |  |  |
|  | 10 d | 283.34 ± 15.85 | 75.07 |  |  |  |  |
|  | **14 d** | **167.96 ± 14.61** | 85.22 |  |  |  |  |
|  | 21 d | 82.69 ± 4.21 | 92.73 |  |  |  |  |
| Clothianidin | 2 h | 20.89 ± 0.34 | - | 200 | 200 | 800 | 150 |
|  | 1 d | 32 ± 1 | - |  |  |  |  |
|  | 3 d | 29.32 ± 1.85 | - |  |  |  |  |
|  | 7 d | 39.79 ± 1.07 | - |  |  |  |  |
|  | **10 d** | **31.98 ± 0.81** | - |  |  |  |  |
|  | 14 d | 25.64 ± 0.42 | - |  |  |  |  |
|  | 21 d | 23.6 ± 0.62 | - |  |  |  |  |
| Trifloxystrobin acid | 2 h | 4.28 ± 0.14 | - | - | - | - | - |
|  | 1 d | 9.97 ± 0.33 | - |  |  |  |  |
|  | 3 d | 14.73 ± 1.45 | - |  |  |  |  |
|  | 7 d | 19.44 ± 0.53 | - |  |  |  |  |
|  | 10 d | 15.83 ± 0.02 | - |  |  |  |  |
|  | 14 d | 18.56 ± 0.59 | - |  |  |  |  |
|  | 21 d | 17.88 ± 0.54 | - |  |  |  |  |

Note: CAC, the Codex Alimentarius Commission; USA, United States of America; EU, European Union. The bolded section indicates the residue level for the recommended pre-harvest interval (PHI).

**Table S5.** Residue levels and processing factors (PFs) of different pesticides during peach processing.

| Pesticides | Processing | Residues (μg/kg) | PFs |
| --- | --- | --- | --- |
| Acetamiprid | raw peaches | 61.48 ± 1.4 | - |
|  | washing (0.5 min) | 48.46 ± 0.68 | 0.79 |
|  | washing (1 min) | 35.85 ± 0.45 | 0.58 |
|  | washing (2 min) | 27.32 ± 1.01 | 0.44 |
|  | washing (5 min) | 28.4 ± 0.56 | 0.46 |
|  | pulps | 16.45 ± 0.61 | 0.27 |
|  | peels | 81.38 ± 2.01 | 1.32 |
|  | crisps | 146.5 ± 7.09 | 2.38 |
| Afidopyropen | raw peaches | 11.23 ± 0.27 | - |
|  | washing (0.5 min) | 8.49 ± 0.45 | 0.76 |
|  | washing (1 min) | 8.59 ± 0.8 | 0.77 |
|  | washing (2 min) | 5.55 ± 0.62 | 0.49 |
|  | washing (5 min) | 3.54 ± 0.11 | 0.32 |
|  | pulps | 2.16 ± 0.26 | 0.19 |
|  | peels | 15.73 ± 2.05 | 1.40 |
|  | crisps | 21.86 ± 1.94 | 1.95 |
| Azoxystrobin | raw peaches | 398.57 ± 10.06 | - |
|  | washing (0.5 min) | 252.67 ± 2.17 | 0.63 |
|  | washing (1 min) | 293.8 ± 5.73 | 0.74 |
|  | washing (2 min) | 164.32 ± 8.4 | 0.41 |
|  | washing (5 min) | 67.41 ± 3.75 | 0.17 |
|  | pulps | 37.98 ± 3.02 | 0.10 |
|  | peels | 568.84 ± 5.72 | 1.43 |
|  | crisps | 1061.98 ± 54.46 | 2.66 |
| Chlorantraniliprole | raw peaches | 416.88 ± 51.56 | - |
|  | washing (0.5 min) | 401.57 ± 58.89 | 0.96 |
|  | washing (1 min) | 245.8 ± 52.41 | 0.59 |
|  | washing (2 min) | 316.29 ± 44.12 | 0.76 |
|  | washing (5 min) | 140.98 ± 21.56 | 0.34 |
|  | pulps | 48.74 ± 7.8 | 0.12 |
|  | peels | 789.72 ± 70.65 | 1.89 |
|  | crisps | 1203.69 ± 115.43 | 2.89 |
| Difenoconazole | raw peaches | 527.73 ± 20.39 | - |
|  | washing (0.5 min) | 431.32 ± 13.51 | 0.82 |
|  | washing (1 min) | 300.49 ± 16.82 | 0.57 |
|  | washing (2 min) | 278.27 ± 12.31 | 0.53 |
|  | washing (5 min) | 174.03 ± 13 | 0.33 |
|  | pulps | 42.07 ± 3.3 | 0.08 |
|  | peels | 1374.1 ± 14.67 | 2.60 |
|  | crisps | 1516.92 ± 120.98 | 2.87 |
| Flonicamid | raw peaches | 187.14 ± 3.08 | - |
|  | washing (0.5 min) | 166.48 ± 3.3 | 0.89 |
|  | washing (1 min) | 123.54 ± 6.07 | 0.66 |
|  | washing (2 min) | 97.23 ± 2.95 | 0.52 |
|  | washing (5 min) | 109.31 ± 2.97 | 0.58 |
|  | pulps | 72.65 ± 3.3 | 0.39 |
|  | peels | 194.34 ± 12.52 | 1.04 |
|  | crisps | 481.39 ± 16.49 | 2.57 |
| Fluopyram | raw peaches | 582.71 ± 7.4 | - |
|  | washing (0.5 min) | 432.68 ± 13.58 | 0.74 |
|  | washing (1 min) | 375.13 ± 17.23 | 0.64 |
|  | washing (2 min) | 254.7 ± 3.28 | 0.44 |
|  | washing (5 min) | 222.97 ± 20.26 | 0.38 |
|  | pulps | 62.51 ± 2.77 | 0.11 |
|  | peels | 852.28 ± 34.52 | 1.46 |
|  | crisps | 1580.29 ± 71.34 | 2.71 |
| Imidacloprid | raw peaches | 473.1 ± 3.71 | - |
|  | washing (0.5 min) | 336.02 ± 11.56 | 0.71 |
|  | washing (1 min) | 291.09 ± 9.61 | 0.62 |
|  | washing (2 min) | 198.67 ± 8.78 | 0.42 |
|  | washing (5 min) | 165.43 ± 5.97 | 0.35 |
|  | pulps | 74.73 ± 2.23 | 0.16 |
|  | peels | 623.69 ± 18.81 | 1.32 |
|  | crisps | 705.74 ± 28.51 | 1.49 |
| Mefentrifluconazole | raw peaches | 701.67 ± 32.11 | - |
|  | washing (0.5 min) | 544.28 ± 6.3 | 0.78 |
|  | washing (1 min) | 438.24 ± 14.34 | 0.62 |
|  | washing (2 min) | 365.8 ± 22.68 | 0.52 |
|  | washing (5 min) | 204.79 ± 10.08 | 0.29 |
|  | pulps | 75.25 ± 3.99 | 0.11 |
|  | peels | 1869.77 ± 20 | 2.66 |
|  | crisps | 1895.59 ± 76.07 | 2.70 |
| Penconazole | raw peaches | 270.32 ± 10.92 | - |
|  | washing (0.5 min) | 263.06 ± 4.21 | 0.97 |
|  | washing (1 min) | 138.52 ± 3.95 | 0.51 |
|  | washing (2 min) | 135.42 ± 7.77 | 0.50 |
|  | washing (5 min) | 109.15 ± 7.19 | 0.40 |
|  | pulps | 22.45 ± 0.3 | 0.08 |
|  | peels | 538.99 ± 23.11 | 1.99 |
|  | crisps | 716.15 ± 29.06 | 2.65 |
| Pymetrozine | raw peaches | 17.81 ± 4 | - |
|  | washing (0.5 min) | 13.51 ± 1.11 | 0.76 |
|  | washing (1 min) | 15.42 ± 2.86 | 0.87 |
|  | washing (2 min) | 5.54 ± 2.27 | 0.31 |
|  | washing (5 min) | 3.88 ± 0.16 | 0.22 |
|  | pulps | 1.42 ± 0.31 | 0.08 |
|  | peels | 32.61 ± 2.5 | 1.83 |
|  | crisps | 27.8 ± 0.66 | 1.56 |
| Pyraclostrobin | raw peaches | 745.01 ± 15.38 | - |
|  | washing (0.5 min) | 639.98 ± 10.77 | 0.86 |
|  | washing (1 min) | 446.01 ± 28.12 | 0.60 |
|  | washing (2 min) | 416.94 ± 13.7 | 0.56 |
|  | washing (5 min) | 280.86 ± 21.39 | 0.38 |
|  | pulps | 63.07 ± 3.24 | 0.08 |
|  | peels | 1458.13 ± 77.12 | 1.96 |
|  | crisps | 1814.17 ± 91.97 | 2.44 |
| Tebuconazole | raw peaches | 410.85 ± 4.32 | - |
|  | washing (0.5 min) | 372.47 ± 12.14 | 0.91 |
|  | washing (1 min) | 258.34 ± 11.93 | 0.63 |
|  | washing (2 min) | 207 ± 7.4 | 0.50 |
|  | washing (5 min) | 161.29 ± 11.92 | 0.39 |
|  | pulps | 44.7 ± 1.72 | 0.11 |
|  | peels | 742.47 ± 26.5 | 1.81 |
|  | crisps | 1265.31 ± 39.35 | 3.08 |
| Thiamethoxam | raw peaches | 90.36 ± 0.39 | - |
|  | washing (0.5 min) | 53.28 ± 0.79 | 0.59 |
|  | washing (1 min) | 64.57 ± 1.1 | 0.71 |
|  | washing (2 min) | 32.63 ± 0.54 | 0.36 |
|  | washing (5 min) | 29.9 ± 0.89 | 0.33 |
|  | pulps | 20.57 ± 0.77 | 0.23 |
|  | peels | 102.78 ± 1.32 | 1.14 |
|  | crisps | 121.43 ± 13.18 | 1.34 |
| Trifloxystrobin | raw peaches | 885.72 ± 29.78 | - |
|  | washing (0.5 min) | 844.03 ± 28.23 | 0.95 |
|  | washing (1 min) | 476.69 ± 23.22 | 0.54 |
|  | washing (2 min) | 602.94 ± 29.68 | 0.68 |
|  | washing (5 min) | 304.36 ± 26.03 | 0.34 |
|  | pulps | 67.01 ± 1.63 | 0.08 |
|  | peels | 1461.01 ± 18.18 | 1.65 |
|  | crisps | 1912.98 ± 134.53 | 2.16 |
| Clothianidin | raw peaches | 32 ± 1 | - |
|  | washing (0.5 min) | 22.27 ± 0.73 | 0.70 |
|  | washing (1 min) | 25.35 ± 1.02 | 0.79 |
|  | washing (2 min) | 17.05 ± 0.3 | 0.53 |
|  | washing (5 min) | 22.06 ± 0.44 | 0.69 |
|  | pulps | 31.01 ± 1.48 | 0.97 |
|  | peels | 42 ± 1.29 | 1.31 |
|  | crisps | 50.49 ± 0.69 | 1.58 |
| Fluopyram benzamide | raw peaches | - | - |
|  | washing (0.5 min) | - | - |
|  | washing (1 min) | - | - |
|  | washing (2 min) | - | - |
|  | washing (5 min) | - | - |
|  | pulps | - | - |
|  | peels | 3.5 ± 0.32 | - |
|  | crisps | 10.79 ± 2.67 | - |
| Trifloxystrobin acid | raw peaches | 9.97 ± 0.33 | - |
|  | washing (0.5 min) | 12.4 ± 0.64 | 1.24 |
|  | washing (1 min) | 6.44 ± 0.35 | 0.65 |
|  | washing (2 min) | 8.98 ± 0.35 | 0.90 |
|  | washing (5 min) | 8.56 ± 0.56 | 0.86 |
|  | pulps | 1.96 ± 0.23 | 0.20 |
|  | peels | 26.63 ± 9.78 | 2.67 |
|  | crisps | 47.82 ± 4.8 | 4.80 |

**Table S6.** Physicochemical parameters of different pesticides used in the study. (molecular weight, MW; solubility in water, Sw, 20°C, mg/ L; melting point, Mp, °C; degradation point, Dp, °C; octanol–water partition coefficient, log Kow, pH = 7, 20°C; vapor pressure, 20°C, mPa).

| Pesticides | Chemical formula | CAS | MW | Sw | Mp | Dp | log Kow | Vp |
| --- | --- | --- | --- | --- | --- | --- | --- | --- |
| Acetamiprid | C₁₀H₁₁ClN₄ | 135410-20-7 | 222.67 | 2950 | 98.9 | 200 | 0.8 | 1.00E-03 |
| Afidopyropen | C₃₃H₃₉NO₉ | 915972-17-7 | 593.7 | 25.1 | 150 | - | 3.45 | 9.99E-03 |
| Azoxystrobin | C₂₂H₁₇N₃O₅ | 131860-33-8 | 403.4 | 6.7 | 116 | 345 | 2.5 | 1.10E-07 |
| Chlorantraniliprole | C₁₈H₁₄BrCl₂N₅O₂ | 500008-45-7 | 483.15 | 0.88 | 209 | 330 | 2.86 | 6.30E-09 |
| Difenoconazole | C₁₉H₁₇Cl₂N₃O₃ | 119446-68-3 | 406.26 | 15 | 82.5 | 337 | 4.36 | 3.33E-05 |
| Flonicamid | C₉H₆F₃N₃O | 158062-67-0 | 229.16 | 5300 | 157.5 | 306 | 0.1 | 2.55E-03 |
| Fluopyram | C₁₆H₁₁ClF₆N₂O | 658066-35-4 | 396.76 | 16 | 117.5 | 300 | 3.3 | 1.20E-03 |
| Imidacloprid | C₉H₁₀ClN₅O₂ | 138261-41-3 | 255.66 | 610 | 144 | 230 | 0.57 | 4.00E-07 |
| Mefentrifluconazole | C₁₈H₁₅ClF₃N₃O₂ | 1417782-03-6 | 397.78 | 0.81 | 126 | 300 | 3.4 | 3.20E-03 |
| Penconazole | C₁₃H₁₅Cl₂N₃ | 66246-88-6 | 284.18 | 73 | 60.3 | - | 3.72 | 3.66E-01 |
| Pymetrozine | C₁₀H₁₁N₅O | 123312-89-0 | 217.23 | 270 | - | 217 | -0.19 | 4.20E-03 |
| Pyraclostrobin | C₁₉H₁₈CIN₃O₄ | 175013-18-0 | 387.82 | 1.9 | 63.7 | 200 | 3.99 | 2.60E-05 |
| Tebuconazole | C₁₆H₂₂ClN₃O | 107534-96-3 | 307.82 | 36 | 105 | 350 | 3.7 | 1.30E-03 |
| Thiamethoxam | C₈H₁₀ClN₅O₃S | 153719-23-4 | 291.71 | 4100 | 139.1 | 147 | -0.13 | 6.60E-06 |
| Trifloxystrobin | C₂₀H₁₉F₃N₂O₄ | 141517-21-7 | 408.37 | 0.61 | 72.9 | 285 | 4.5 | 3.40E-03 |

**Table S7.** The estimated daily intake (EDI), estimation of short term intake (ESTI), chronic risk quotient (RQc) and acute risk quotient (RQa) values of different pesticides for children and the general populations from peach consumption.

| Risk parameters | Pesticides | Time | | | | | | |
| --- | --- | --- | --- | --- | --- | --- | --- | --- |
|  |  | 2 h | 1 d | 3 d | 7 d | 10 d | 14 d | 21 d |
| EDI (children) | Acetamiprid | 0.0738 | 0.0329 | 0.0291 | 0.0192 | 0.0172 | 0.0116 | 0.0095 |
|  | Afidopyropen | 0.0143 | 0.0060 | 0.0057 | 0.0026 | 0.0017 | 0.0011 | 0.0011 |
|  | Azoxystrobin | 0.3060 | 0.2116 | 0.1952 | 0.1316 | 0.0663 | 0.0244 | 0.0180 |
|  | Chlorantraniliprole | 0.2534 | 0.2213 | 0.1978 | 0.1547 | 0.1429 | 0.1041 | 0.0536 |
|  | Clothianidin | 0.0111 | 0.0170 | 0.0156 | 0.0211 | 0.0170 | 0.0136 | 0.0125 |
|  | Difenoconazole | 0.3817 | 0.2802 | 0.2406 | 0.1585 | 0.1469 | 0.0744 | 0.0443 |
|  | Flonicamid | 0.1952 | 0.0994 | 0.0876 | 0.0562 | 0.0476 | 0.0346 | 0.0234 |
|  | Fluopyram | 0.3100 | 0.3060 | 0.1831 | 0.1554 | 0.0838 | 0.0763 | 0.0006 |
|  | Imidacloprid | 0.6257 | 0.2512 | 0.1941 | 0.1167 | 0.0934 | 0.0556 | 0.0390 |
|  | Mefentrifluconazole | 0.5415 | 0.3726 | 0.3756 | 0.2877 | 0.2145 | 0.1106 | 0.0711 |
|  | Penconazole | 0.2552 | 0.1435 | 0.1213 | 0.0632 | 0.0435 | 0.0157 | 0.0055 |
|  | Pymetrozine | 0.0411 | 0.0095 | 0.0046 | 0.0033 | 0.0014 | 0.0009 | 0.0009 |
|  | Pyraclostrobin | 0.4874 | 0.3956 | 0.3344 | 0.2362 | 0.1975 | 0.1126 | 0.0765 |
|  | Tebuconazole | 0.4292 | 0.2181 | 0.2064 | 0.1202 | 0.0935 | 0.0368 | 0.0185 |
|  | Thiamethoxam | 0.1547 | 0.0480 | 0.0344 | 0.0147 | 0.0054 | 0.0029 | 0.0017 |
|  | Trifloxystrobin | 0.6083 | 0.4814 | 0.4399 | 0.2520 | 0.1681 | 0.1099 | 0.0638 |
| RQc (children) | Acetamiprid | 0.0011 | 0.0005 | 0.0004 | 0.0003 | 0.0002 | 0.0002 | 0.0001 |
|  | Afidopyropen | 0.0002 | 0.0001 | 0.0001 | 0.0000 | 0.0000 | 0.0000 | 0.0000 |
|  | Azoxystrobin | 0.0015 | 0.0011 | 0.0010 | 0.0007 | 0.0003 | 0.0001 | 0.0001 |
|  | Chlorantraniliprole | 0.0001 | 0.0001 | 0.0001 | 0.0001 | 0.0001 | 0.0001 | 0.0000 |
|  | Clothianidin | 0.0001 | 0.0002 | 0.0002 | 0.0002 | 0.0002 | 0.0001 | 0.0001 |
|  | Difenoconazole | 0.0382 | 0.0280 | 0.0241 | 0.0158 | 0.0147 | 0.0074 | 0.0044 |
|  | Flonicamid | 0.0028 | 0.0014 | 0.0013 | 0.0008 | 0.0007 | 0.0005 | 0.0003 |
|  | Fluopyram | 0.0310 | 0.0306 | 0.0183 | 0.0155 | 0.0084 | 0.0076 | 0.0001 |
|  | Imidacloprid | 0.0104 | 0.0042 | 0.0032 | 0.0019 | 0.0016 | 0.0009 | 0.0007 |
|  | Mefentrifluconazole | 0.0155 | 0.0106 | 0.0107 | 0.0082 | 0.0061 | 0.0032 | 0.0020 |
|  | Penconazole | 0.0085 | 0.0048 | 0.0040 | 0.0021 | 0.0014 | 0.0005 | 0.0002 |
|  | Pymetrozine | 0.0014 | 0.0003 | 0.0002 | 0.0001 | 0.0000 | 0.0000 | 0.0000 |
|  | Pyraclostrobin | 0.0162 | 0.0132 | 0.0111 | 0.0079 | 0.0066 | 0.0038 | 0.0026 |
|  | Tebuconazole | 0.0143 | 0.0073 | 0.0069 | 0.0040 | 0.0031 | 0.0012 | 0.0006 |
|  | Thiamethoxam | 0.0019 | 0.0006 | 0.0004 | 0.0002 | 0.0001 | 0.0000 | 0.0000 |
|  | Trifloxystrobin | 0.0152 | 0.0120 | 0.0110 | 0.0063 | 0.0042 | 0.0027 | 0.0016 |
| EDI (general population) | Acetamiprid | 0.0171 | 0.0076 | 0.0067 | 0.0044 | 0.0040 | 0.0027 | 0.0022 |
|  | Afidopyropen | 0.0033 | 0.0014 | 0.0013 | 0.0006 | 0.0004 | 0.0003 | 0.0002 |
|  | Azoxystrobin | 0.0708 | 0.0490 | 0.0452 | 0.0305 | 0.0153 | 0.0057 | 0.0042 |
|  | Chlorantraniliprole | 0.0586 | 0.0512 | 0.0458 | 0.0358 | 0.0331 | 0.0241 | 0.0124 |
|  | Clothianidin | 0.0026 | 0.0039 | 0.0036 | 0.0049 | 0.0039 | 0.0031 | 0.0029 |
|  | Difenoconazole | 0.0883 | 0.0648 | 0.0557 | 0.0367 | 0.0340 | 0.0172 | 0.0102 |
|  | Flonicamid | 0.0452 | 0.0230 | 0.0203 | 0.0130 | 0.0110 | 0.0080 | 0.0054 |
|  | Fluopyram | 0.0717 | 0.0708 | 0.0424 | 0.0360 | 0.0194 | 0.0176 | 0.0001 |
|  | Imidacloprid | 0.1448 | 0.0581 | 0.0449 | 0.0270 | 0.0216 | 0.0129 | 0.0090 |
|  | Mefentrifluconazole | 0.1253 | 0.0862 | 0.0869 | 0.0665 | 0.0496 | 0.0256 | 0.0164 |
|  | Penconazole | 0.0590 | 0.0332 | 0.0281 | 0.0146 | 0.0101 | 0.0036 | 0.0013 |
|  | Pymetrozine | 0.0095 | 0.0022 | 0.0011 | 0.0008 | 0.0003 | 0.0002 | 0.0002 |
|  | Pyraclostrobin | 0.1128 | 0.0915 | 0.0774 | 0.0546 | 0.0457 | 0.0260 | 0.0177 |
|  | Tebuconazole | 0.0993 | 0.0505 | 0.0477 | 0.0278 | 0.0216 | 0.0085 | 0.0043 |
|  | Thiamethoxam | 0.0358 | 0.0111 | 0.0080 | 0.0034 | 0.0012 | 0.0007 | 0.0004 |
|  | Trifloxystrobin | 0.1407 | 0.1114 | 0.1018 | 0.0583 | 0.0389 | 0.0254 | 0.0148 |
| RQc (general population) | Acetamiprid | 0.0002 | 0.0001 | 0.0001 | 0.0001 | 0.0001 | 0.0000 | 0.0000 |
|  | Afidopyropen | 0.0000 | 0.0000 | 0.0000 | 0.0000 | 0.0000 | 0.0000 | 0.0000 |
|  | Azoxystrobin | 0.0004 | 0.0002 | 0.0002 | 0.0002 | 0.0001 | 0.0000 | 0.0000 |
|  | Chlorantraniliprole | 0.0000 | 0.0000 | 0.0000 | 0.0000 | 0.0000 | 0.0000 | 0.0000 |
|  | Clothianidin | 0.0000 | 0.0000 | 0.0000 | 0.0000 | 0.0000 | 0.0000 | 0.0000 |
|  | Difenoconazole | 0.0088 | 0.0065 | 0.0056 | 0.0037 | 0.0034 | 0.0017 | 0.0010 |
|  | Flonicamid | 0.0006 | 0.0003 | 0.0003 | 0.0002 | 0.0002 | 0.0001 | 0.0001 |
|  | Fluopyram | 0.0072 | 0.0071 | 0.0042 | 0.0036 | 0.0019 | 0.0018 | 0.0000 |
|  | Imidacloprid | 0.0024 | 0.0010 | 0.0007 | 0.0004 | 0.0004 | 0.0002 | 0.0002 |
|  | Mefentrifluconazole | 0.0036 | 0.0025 | 0.0025 | 0.0019 | 0.0014 | 0.0007 | 0.0005 |
|  | Penconazole | 0.0020 | 0.0011 | 0.0009 | 0.0005 | 0.0003 | 0.0001 | 0.0000 |
|  | Pymetrozine | 0.0003 | 0.0001 | 0.0000 | 0.0000 | 0.0000 | 0.0000 | 0.0000 |
|  | Pyraclostrobin | 0.0038 | 0.0031 | 0.0026 | 0.0018 | 0.0015 | 0.0009 | 0.0006 |
|  | Tebuconazole | 0.0033 | 0.0017 | 0.0016 | 0.0009 | 0.0007 | 0.0003 | 0.0001 |
|  | Thiamethoxam | 0.0004 | 0.0001 | 0.0001 | 0.0000 | 0.0000 | 0.0000 | 0.0000 |
|  | Trifloxystrobin | 0.0035 | 0.0028 | 0.0025 | 0.0015 | 0.0010 | 0.0006 | 0.0004 |
| ESTI (children) | Acetamiprid | 7.32 | 3.26 | 2.89 | 1.90 | 1.70 | 1.15 | 0.94 |
|  | Afidopyropen | 1.42 | 0.59 | 0.57 | 0.26 | 0.16 | 0.11 | 0.11 |
|  | Azoxystrobin | 30.33 | 20.98 | 19.36 | 13.05 | 6.57 | 2.42 | 1.79 |
|  | Chlorantraniliprole | 25.13 | 21.95 | 19.61 | 15.34 | 14.17 | 10.32 | 5.32 |
|  | Clothianidin | 1.10 | 1.68 | 1.54 | 2.09 | 1.68 | 1.35 | 1.24 |
|  | Difenoconazole | 37.84 | 27.78 | 23.85 | 15.71 | 14.57 | 7.38 | 4.39 |
|  | Flonicamid | 19.36 | 9.85 | 8.68 | 5.57 | 4.71 | 3.43 | 2.32 |
|  | Fluopyram | 30.73 | 30.34 | 18.15 | 15.41 | 8.31 | 7.56 | 0.06 |
|  | Imidacloprid | 62.04 | 24.91 | 19.25 | 11.57 | 9.26 | 5.51 | 3.87 |
|  | Mefentrifluconazole | 53.69 | 36.94 | 37.24 | 28.52 | 21.26 | 10.97 | 7.05 |
|  | Penconazole | 25.30 | 14.23 | 12.03 | 6.26 | 4.31 | 1.55 | 0.55 |
|  | Pymetrozine | 4.08 | 0.94 | 0.46 | 0.32 | 0.14 | 0.09 | 0.09 |
|  | Pyraclostrobin | 48.32 | 39.22 | 33.16 | 23.42 | 19.58 | 11.16 | 7.59 |
|  | Tebuconazole | 42.56 | 21.63 | 20.46 | 11.92 | 9.27 | 3.65 | 1.83 |
|  | Thiamethoxam | 15.34 | 4.76 | 3.41 | 1.46 | 0.53 | 0.29 | 0.17 |
|  | Trifloxystrobin | 60.32 | 47.73 | 43.61 | 24.98 | 16.67 | 10.89 | 6.33 |
| RQa (children) | Acetamiprid | 0.0732 | 0.0326 | 0.0289 | 0.0190 | 0.0170 | 0.0115 | 0.0094 |
|  | Afidopyropen | - | - | - | - | - | - | - |
|  | Azoxystrobin | - | - | - | - | - | - | - |
|  | Chlorantraniliprole | - | - | - | - | - | - | - |
|  | Clothianidin | 0.0018 | 0.0028 | 0.0026 | 0.0035 | 0.0028 | 0.0022 | 0.0021 |
|  | Difenoconazole | 0.1261 | 0.0926 | 0.0795 | 0.0524 | 0.0486 | 0.0246 | 0.0146 |
|  | Flonicamid | - | - | - | - | - | - | - |
|  | Fluopyram | 0.0615 | 0.0607 | 0.0363 | 0.0308 | 0.0166 | 0.0151 | 0.0001 |
|  | Imidacloprid | 0.1551 | 0.0623 | 0.0481 | 0.0289 | 0.0232 | 0.0138 | 0.0097 |
|  | Mefentrifluconazole | 0.3579 | 0.2463 | 0.2483 | 0.1901 | 0.1418 | 0.0731 | 0.0470 |
|  | Penconazole | 0.0316 | 0.0178 | 0.0150 | 0.0078 | 0.0054 | 0.0019 | 0.0007 |
|  | Pymetrozine | 0.0408 | 0.0094 | 0.0046 | 0.0032 | 0.0014 | 0.0009 | 0.0009 |
|  | Pyraclostrobin | 0.9665 | 0.7844 | 0.6632 | 0.4684 | 0.3916 | 0.2232 | 0.1517 |
|  | Tebuconazole | 0.1419 | 0.0721 | 0.0682 | 0.0397 | 0.0309 | 0.0122 | 0.0061 |
|  | Thiamethoxam | 0.0153 | 0.0048 | 0.0034 | 0.0015 | 0.0005 | 0.0003 | 0.0002 |
|  | Trifloxystrobin | - | - | - | - | - | - | - |
| ESTI (general population) | Acetamiprid | 2.52 | 1.12 | 0.99 | 0.66 | 0.59 | 0.40 | 0.32 |
|  | Afidopyropen | 0.49 | 0.20 | 0.19 | 0.09 | 0.06 | 0.04 | 0.04 |
|  | Azoxystrobin | 10.44 | 7.22 | 6.66 | 4.49 | 2.26 | 0.83 | 0.62 |
|  | Chlorantraniliprole | 8.65 | 7.55 | 6.75 | 5.28 | 4.88 | 3.55 | 1.83 |
|  | Clothianidin | 0.38 | 0.58 | 0.53 | 0.72 | 0.58 | 0.46 | 0.43 |
|  | Difenoconazole | 13.02 | 9.56 | 8.21 | 5.41 | 5.01 | 2.54 | 1.51 |
|  | Flonicamid | 6.66 | 3.39 | 2.99 | 1.92 | 1.62 | 1.18 | 0.80 |
|  | Fluopyram | 10.58 | 10.44 | 6.25 | 5.30 | 2.86 | 2.60 | 0.02 |
|  | Imidacloprid | 21.35 | 8.57 | 6.62 | 3.98 | 3.19 | 1.90 | 1.33 |
|  | Mefentrifluconazole | 18.48 | 12.71 | 12.82 | 9.82 | 7.32 | 3.78 | 2.42 |
|  | Penconazole | 8.71 | 4.90 | 4.14 | 2.16 | 1.48 | 0.53 | 0.19 |
|  | Pymetrozine | 1.40 | 0.32 | 0.16 | 0.11 | 0.05 | 0.03 | 0.03 |
|  | Pyraclostrobin | 16.63 | 13.50 | 11.41 | 8.06 | 6.74 | 3.84 | 2.61 |
|  | Tebuconazole | 14.65 | 7.44 | 7.04 | 4.10 | 3.19 | 1.26 | 0.63 |
|  | Thiamethoxam | 5.28 | 1.64 | 1.17 | 0.50 | 0.18 | 0.10 | 0.06 |
|  | Trifloxystrobin | 20.76 | 16.43 | 15.01 | 8.60 | 5.74 | 3.75 | 2.18 |
| RQa (general population) | Acetamiprid | 0.0252 | 0.0112 | 0.0099 | 0.0066 | 0.0059 | 0.0040 | 0.0032 |
|  | Afidopyropen | - | - | - | - | - | - | - |
|  | Azoxystrobin | - | - | - | - | - | - | - |
|  | Chlorantraniliprole | - | - | - | - | - | - | - |
|  | Clothianidin | 0.0006 | 0.0010 | 0.0009 | 0.0012 | 0.0010 | 0.0008 | 0.0007 |
|  | Difenoconazole | 0.0434 | 0.0319 | 0.0274 | 0.0180 | 0.0167 | 0.0085 | 0.0050 |
|  | Flonicamid | - | - | - | - | - | - | - |
|  | Fluopyram | 0.0212 | 0.0209 | 0.0125 | 0.0106 | 0.0057 | 0.0052 | 0.0000 |
|  | Imidacloprid | 0.0534 | 0.0214 | 0.0166 | 0.0100 | 0.0080 | 0.0047 | 0.0033 |
|  | Mefentrifluconazole | 0.1232 | 0.0848 | 0.0855 | 0.0654 | 0.0488 | 0.0252 | 0.0162 |
|  | Penconazole | 0.0109 | 0.0061 | 0.0052 | 0.0027 | 0.0019 | 0.0007 | 0.0002 |
|  | Pymetrozine | 0.0140 | 0.0032 | 0.0016 | 0.0011 | 0.0005 | 0.0003 | 0.0003 |
|  | Pyraclostrobin | 0.3326 | 0.2700 | 0.2283 | 0.1612 | 0.1348 | 0.0768 | 0.0522 |
|  | Tebuconazole | 0.0488 | 0.0248 | 0.0235 | 0.0137 | 0.0106 | 0.0042 | 0.0021 |
|  | Thiamethoxam | 0.0053 | 0.0016 | 0.0012 | 0.0005 | 0.0002 | 0.0001 | 0.0001 |
|  | Trifloxystrobin | - | - | - | - | - | - | - |
